# Obscure Severe Infrarenal Aortoiliac Stenosis With Severe Transient Lactic Acidosis

**DOI:** 10.1177/2324709613479940

**Published:** 2013-01-01

**Authors:** Teerapat Nantsupawat, Charoen Mankongpaisarnrung, Suthipong Soontrapa, Chok Limsuwat, Kenneth Nugent

**Affiliations:** 1 Texas Tech University Health Sciences Center, Lubbock, TX, USA

**Keywords:** infrarenal, aortoiliac stenosis, transient, lactic acidosis, limb ischemia

## Abstract

A 57-year-old man presented with sudden onset of leg pain, right-sided weakness, aphasia, confusion, drooling, and severe lactic acidosis (15 mmol/L). He had normal peripheral pulses and demonstrated no pain, pallor, poikilothermia, paresthesia, or paralysis. Empiric antibiotics, aspirin, full-dose enoxaparin, and intravenous fluid were initiated. Lactic acid level decreased to 2.5 mmol/L. The patient was subsequently extubated and was alert and oriented with no complaints of leg or abdominal pain. Unexpectedly, the patient developed cardiac arrest, rebound severe lactic acidosis (8.13 mmol/L), and signs of acute limb ischemia. Emergent computed tomography of the aorta confirmed infrarenal aortoiliac thrombosis. Transient leg pain and transient severe lactic acidosis can be unusual presentations of severe infrarenal aortoiliac stenosis. When in doubt, vascular studies should be implemented without delay to identify this catastrophic diagnosis.

## Introduction

Acute abdominal aortic occlusion is rare and is associated with high morbidity and mortality rates. With the extensive use of newer endovascular techniques, most published series report a 10% to 30% 30-day amputation rate.^1^ Postsurgical revascularization mortality as high as 14% to 60% has been reported.^2^ Acute aortic occlusion can result from thrombus formation, saddle embolism, false-lumen expansion in aortic dissection, aortic trauma, and other etiologies related to arteriosclerosis or hypercoagulability.^2^ Symptoms and signs of patients vary depending on the level of occlusion. Patients with severe infrarenal aortic stenosis can present with acute limb ischemia, manifested by sudden pain, pallor, pulselessness, paresthesia, and paralysis (the 5 Ps). Bowel ischemia can also be present due to impaired circulation to the inferior mesenteric artery. Laboratory tests typically reveal elevated serum lactate levels produced from ischemic tissue, causing lactic acidosis.

Lactic acidosis is defined as an elevated anion gap metabolic acidosis with a serum lactate concentration more than 4 mmol/L. Lactic acid is formed by the reduction of pyruvate by lactate dehydrogenase during anaerobic metabolism. Lactic acidosis thus usually implies tissue hypoxia or impaired aerobic metabolism in mitochondria.^3^ If the conditions that increase lactate production or decrease lactate utilization during an acute illness resolve, serum lactate can rapidly normalize within hours. Grand mal seizures, strenuous exercise, propofol infusion syndrome, alcoholic intoxication with thiamine deficiency, high-dose metformin, and repeated intravenous infusions of sorbitol and ethanol have been reported as causes of transient or reversible lactic acidosis.^4-9^ Unlike the temporary processes mentioned above, acute aortic occlusion and acute limb ischemia once occurred usually persist or progress unless catheter-based or surgical revascularization occurs. We present a patient with multi-organ dysfunction, including acute kidney injury, hepatitis, and encephalopathy; associated symptoms included transient leg pain and lactic acidosis, which resolved within the first 24 hours after empiric antibiotics and supportive measures. Later, the patient developed recurrent leg pain with severe lactic acidosis; computed tomography angiography revealed an acute infrarenal aortic high-grade stenosis.

## Case Description

A 57-year-old man with a history of hypertension and right hip fracture called emergency medical services and complained of severe leg pain. When the emergency medical services team arrived on scene, the patient was found down on the floor with right leg weakness; he was confused and aphasic. No fumes or smoke was evident. His initial laboratory investigation at a local hospital showed potassium 6.1 mEq/L, blood urea nitrogen 26 mg/dL, creatinine 2.3 mg/dL, D-dimer 2240 µg/L, lactic acid 15 mmol/L, and glucose 166 mg/dL. Arterial blood gas analysis disclosed pH 6.60, PCO_2_ 50 mm Hg, Po_2_ 176 mm Hg, and HCO_3_ 4.9 mEq/L on a Fio_2_ 100%. Due to altered mental status, he was intubated and was subsequently transferred to our medical facility. On route, for the treatment of hyperkalemia, he was given 3 ampoules of sodium bicarbonate, calcium gluconate, kayexalate, and insulin with dextrose water.

Six hours after the incident, the patient was more alert and oriented and his leg pain had subsided. Details of smoking history could not be obtained due to the intubation. On physical examination, temperature was 35.8°C, heart rate 107 bpm, blood pressure 137/94 mm Hg, and respiratory rate 14 breaths/minute. He had regular heart rhythm, no murmur or pericardial rub. His abdomen was soft with active bowel sound. There were no symptoms and signs of limb ischemia on admission. He had no leg pain, pallor, poikilothermia, pulselessness, paresthesia, or paralysis. Motor power was at least grade 4 in all 4 extremities. His dorsalis pedis pulse was 2+ bilaterally. Initial investigations at our medical facility showed white blood cell 11400/µL, neutrophils 89%, hemoglobin 13.1 g/dL, hematocrit 38.6%, platelet 159000/µL, blood urea nitrogen 29 mg/dL, creatinine 2.0 mg/dL, sodium 142 mEq/L, potassium 3.9 mEq/L, chloride 102 mEq/L, bicarbonate 19 mEq/L, lactic acid 4.25 mmol/L, troponin T 0.1 ng/mL, and negative for acetone. Electrocardiogram showed sinus tachycardia and no ST-T change. The liver function test demonstrated aspartate aminotransferase 1697 IU/L, alanine aminotransferase 1263 IU/L, alkaline phosphatase 138 IU/L, total bilirubin 0.7 mg/dL, total protein 6.2 gm/dL, and albumin 3.5 mg/dL. Urinalysis showed white blood cell 5 to 10/high-power field, red blood cell 0 to 3/high-power field, moderate blood but negative for urine myoglobin. Coagulogram showed prothrombin time 17.5 seconds, partial thromboplastin time 25.8 seconds, and international normalized ratio 1.59. Calculated fractional excretion of sodium and urea were 2.11% and 43.67%, respectively, consistent with acute tubular necrosis. The viral hepatitis panel was negative except for a positive AntiHBc IgG antibody. Arterial blood gas analysis on admission depicted pH 7.319, PO_2_ 130.3 mm Hg, PCO_2_ 40.8 mm Hg, HCO_3_ 20.5 mEq/L, and Sao_2_ 100%, carboxyhemoglobin 0.2%, and methemoglobin 0.1% while on assist-control mode of mechanical ventilation with Fio_2_ 100%. Computed tomography of head was performed and showed chronic right basal ganglion infarct. Transthoracic echocardiography was performed and showed severely depressed left ventricular function with ejection fraction of less than 20%, grade II/IV diastolic dysfunction with mild TR, mild MR, and mild PR. Akinesis of the septal wall and hypokinesis of the anterolateral wall were present. Moreover, computed tomography of abdomen, pelvis, and chest without contrast revealed right lower lung consolidation with small right pleural effusion, moderately bilateral nonspecific perinephric fat stranding, submucosal fatty infiltration, and mild wall thickening of the descending colon down to the rectum. Subtle perirectal fat stranding was also visualized. Initially, he was treated with empiric antibiotics for possible pneumonia with severe sepsis with piperacillin-tazobactam, levofloxacin, and vancomycin. Aspirin and full-dose enoxaparin were started to cover possible acute coronary syndrome. Surgery was consulted due to a concern of bowel ischemia. Proctoscopy was done and showed normal pink rectal mucosa without blood in the vault. Because the patient was hemodynamically stable, had no abdominal pain, and lactic acid level had returned to normal, the surgery service recommended continued fluid replacement for volume expansion.

**Figure 1.**
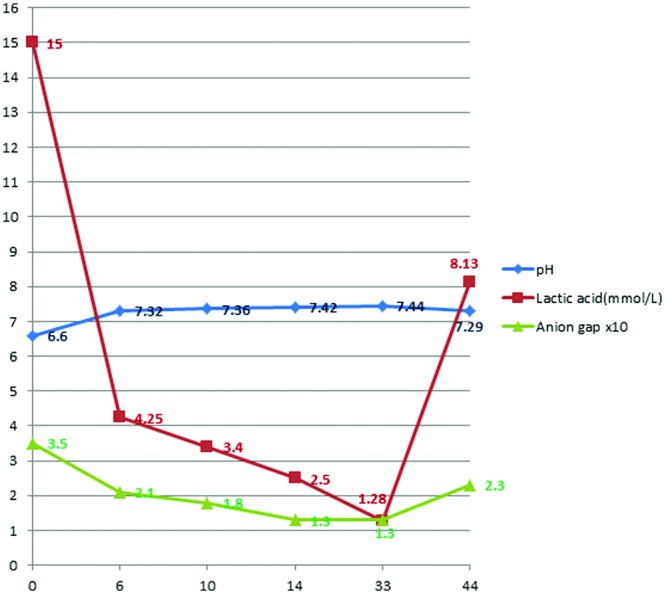
pH, lactic acid level, and anion gap trends during hospitalization.

**Figure 2.**
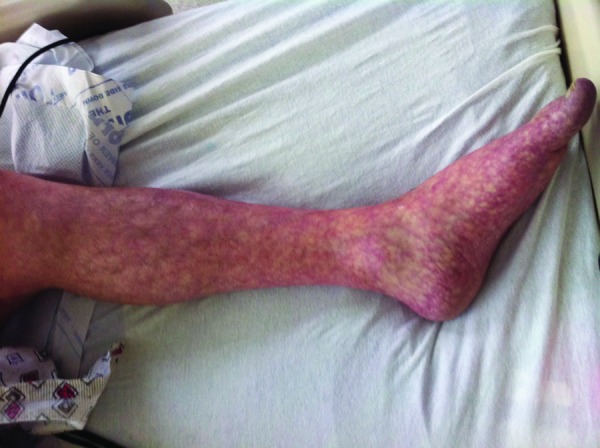
Livedo reticularis of left leg.

**Figure 3.**
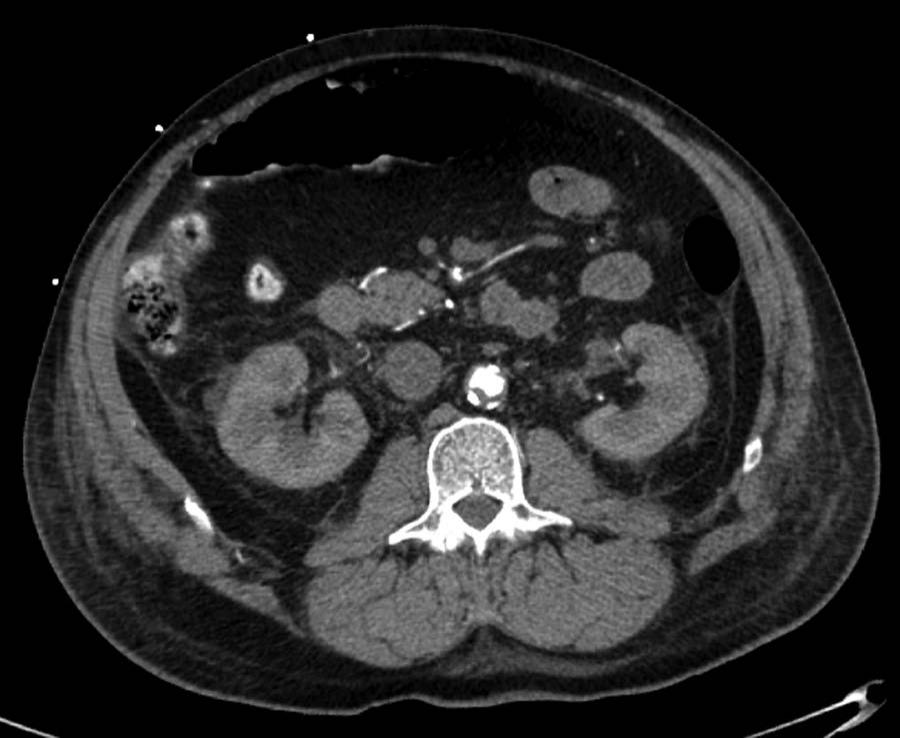
Computed tomography angiogram of aorta demonstrating intraluminal thrombus of abdominal aorta.

**Figure 4.**
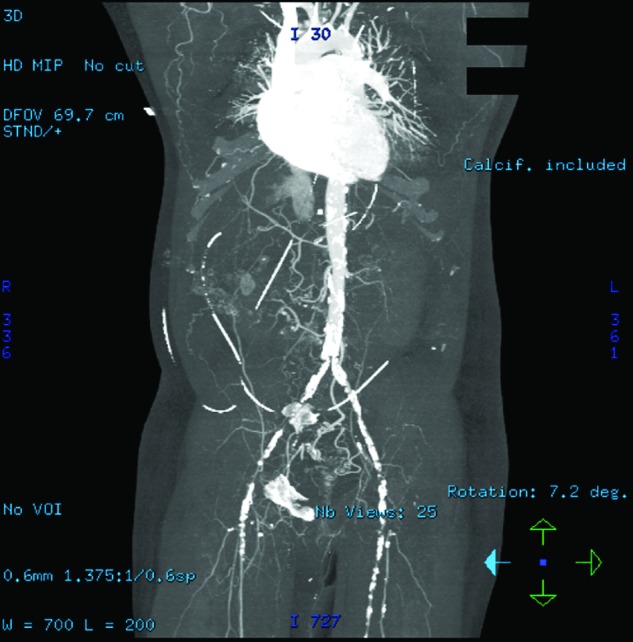
Three-dimensional computed tomography angiogram reconstruction demonstrating extensive severe luminal narrowing of the infrarenal abdominal aorta, bilateral common iliac, external iliac, and common femoral arteries by calcified and noncalcified plaques.

His lactate levels decreased spontaneously from 15 mmol/L to 3.39, 2.52, and 1.28 mmol/L at 0, 10, 14, and 33 hours, respectively, after his initial symptoms, along with an improvement of arterial pH and anion gap (Figure 1). After 24 hours of admission, he improved dramatically and was extubated on day 2 of admission. Five hours after extubation (42 hours after admission), he started to have left leg pain. His left leg turned bluish and became paler with prominent livedo reticularis throughout his left leg (Figure 2). Therefore, a heparin drip was initiated promptly and vascular surgery was consulted. Soon after that, the patient developed sudden cardiac arrest with pulseless electrical activity. He had return of spontaneous circulation after 15 minutes of cardiopulmonary resuscitation. At that moment, his lactate level rebounded to 8.13 mmol/L. To rule out acute aortic dissection and acute massive pulmonary embolism as well as acute left limb gangrene from embolism, computed tomography of pulmonary artery with contrast and computed tomography angiogram of aorta were performed. He had no pulmonary emboli or abdominal aortic dissection. He did have high-grade stenosis of the infrarenal abdominal aorta just above the bifurcation, near total occlusion of the right common iliac artery, severe luminal narrowing of the left common iliac artery, bilateral external iliac arteries, and bilateral common femoral arteries by calcified and noncalcified plaques (Figures 3 and 4). Emergent revascularization was planned but the patient developed pulseless electrical activity/asystole again and passed away.

## Discussion

This patient initially presented with sudden onset of leg pain, right-sided weakness, aphasia, confusion, and drooling. This presentation suggested a cerebrovascular accident. However, motor power at least grade 4 in all 4 extremities and no infarction or intracranial hemorrhage on a computed tomography of the brain made this diagnosis unlikely. The patient also presented with severe lactic acidosis. The causes of lactic acidosis can be divided into those associated with systemic hypoperfusion or impaired tissue oxygenation (type A) and those without tissue hypoxia (type B). Type B lactic acidosis can be divided into B1 (related to underlying diseases, eg, renal failure, hepatic failure, malignancy, HIV), B2 (related to drugs and toxins), and B3 (related to inborn errors of metabolism).^3^ In our case, lactic acidosis could be from grand mal seizure, renal failure, hepatic failure, congestive heart failure from acute coronary syndrome, severe stifis, limb ischemia, or bowel ischemia. Limb ischemia was unlikely at the beginning because the patient had normal peripheral pulses and demonstrated no pain, pallor, poikilothermia, paresthesia, or paralysis. Since the patient’s clinical status and severe lactic acidosis dramatically improved after empiric antibiotics, aspirin, full-dose enoxaparin, and supportive measures with intravenous fluid, more detailed investigations, such as coronary angiography, computed tomography angiogram of mesenteric arteries, were deferred until the patient was more stable and at less risk for contrast-induced nephropathy. Thirty-six hours after the incident, the patient was extubated; he was alert and oriented with no complaints of leg or abdominal pain. Unexpectedly, the patient developed cardiac arrest, rebound severe lactic acidosis, and signs of acute limb ischemia. Emergent computed tomography of the aorta confirmed infrarenal aortic thrombosis.

On review, the first peak of severe lactic acidosis in this patient was most likely from acute infrarenal aortic occlusion causing acute limb ischemia and bowel ischemia. However, by the time patient arrived at our facility approximately 6 hours after onset, he had no leg pain, no signs of the 5 Ps, and his lactic acid level was already lower. The dramatic improvement obscured symptoms and signs of severe infrarenal aortic stenosis and acute limb ischemia in our patient; we missed an opportunity to make an early diagnosis and urgent revascularization. Aspirin and full-dose enoxaparin might have prevented further embolic and thrombus propagation. Mechanical ventilation and intravenous fluid, which provided cardiopulmonary support, could improve blood flow through a partially occluded infrarenal aorta to lower extremities. An unstable thrombus, without revascularization, finally propagated to total occlusion of aorta and its branches. There was a recent case series that proved the success of nonoperative treatment with heparin for acute limb ischemia in infants.^10^ But in adults, there is minimal evidence to support nonoperative treatment in acute aortic occlusion or acute limb ischemia. The standard treatment is urgent or emergent endovascular thrombolysis, endovascular thrombectomy, or surgical revascularization.^1^

A marked elevation of liver enzymes can be caused by acute viral hepatitis, drugs/toxins, or hypoxic hepatitis (shock liver). The patient had a negative viral hepatitis profile and no potential drugs/toxins were identified. Acute tubular necrosis along with elevated liver enzymes makes hypoxic hepatitis likely in this patient. It is interesting that acute lower limb ischemia can be the triggering condition for hypoxic ischemic hepatitis as reported in one prospective case series.^11^ The pathologic mechanism is believed to be from a release of inflammatory mediators and cellular debris from ischemic myocytes and digestive tract during ischemic phase that activate Kupffer cell to release cytokines and reactive oxygen, causing neutrophils migration and more damage to liver cells.^11^ The severely depressed myocardial function in our patient could have also contributed to hypoxic hepatitis by decreased oxygen delivery and impaired oxygen diffusion in congested liver.^12,13^

Our patient has infrarenal aortoiliac high-grade stenosis, diffuse disease involving bilateral common iliac arteries, external iliac arteries, and common femoral arteries. This is consistent with a type D lesion according to TASC classification of aortoiliac lesions.^1^ Surgery is the treatment of choice for type D lesions. For critical limb ischemia, aortobifemoral bypass has 5-year and 10-year patency rates of 80% and 72%, respectively.^14^ Axillo-bifemoral bypass and femoro-femoral bypass has 5-year patency rates of 71% and 75%, respectively. After revascularization, a patient requires close monitoring for “reperfusion injury.” Reperfusion injury causes renal, pulmonary, liver, and cardiac complications from inflammatory mediators and cytokines that are released from reperfused ischemic muscles. In addition, a compartment syndrome and rhabdomyolysis, which requires emergent fasciotomy and aggressive hydration, can occur from capillary fluid leakage to the muscle compartment.^15^

In summary, this case provides 2 important messages: (*a*) Grand mal seizures, strenuous exercise, propofol infusion syndrome, alcoholic intoxication with thiamine deficiency, high-dose metformin, and repeated intravenous infusions of sorbitol/ethanol can cause reversible lactic acidosis. Acute arterial occlusion rarely can present with profound transient severe lactic acidosis without signs of persistent limb ischemia. Thus, it should be included in the differential diagnosis of transient lactic acidosis and requires thorough urgent investigation. (*b*) Acute severe aortoiliac stenosis and acute limb ischemia can be obscured and initially improved by anticoagulation and conservative measures. When in doubt, vascular studies to identify this catastrophic diagnosis should be implemented without delay.
